# Impact of Photo-Excitation on Leakage Current and Negative Bias Instability in InSnZnO Thickness-Varied Thin-Film Transistors

**DOI:** 10.3390/nano10091782

**Published:** 2020-09-09

**Authors:** Dapeng Wang, Mamoru Furuta, Shigekazu Tomai, Koki Yano

**Affiliations:** 1 Key Laboratory of Applied Surface and Colloid Chemistry, Ministry of Education, Shaanxi Normal University, Xian 710119, China; 2 Shaanxi Key Laboratory for Advanced Energy Devices; Shaanxi Engineering Lab for Advanced Energy Technology, Shaanxi Normal University, Xian 710119, China; 3 School of Materials Science and Engineering, Shaanxi Normal University, Xian 710119, China; 4 School of Environmental Science and Engineering, Kochi University of Technology, Kami, Kochi 782-8502, Japan; 5 Center for Nanotechnology in Research Institute, Kochi University of Technology, Kami, Kochi 782-8502, Japan; 6 Advanced Technology Research Laboratories, Idemitsu Kosan Co. Ltd., Sodegaura, Chiba 299-0293, Japan; shigekazu.tomai.6560@idss.co.jp (S.T.); koki.yano@idemitsu.com (K.Y.)

**Keywords:** photo-excitation, leakage current, NBIS-induced instability, InSnZnO thickness, oxide TFTs

## Abstract

InSnZnO thin-film transistors (ITZO TFTs), having high carrier mobility, guarantee the benefits of potential applications in the next generation of super-high-definition flat-panel displays. However, the impact of photo-excitation on the leakage current and negative bias stress (NBIS) of ITZO TFTs must be further explored. In this study, the ITZO thickness (*T*_ITZO_) is designed to tailor the initial performance of devices, especially for the 100 nm *T*_ITZO_ TFT, producing excellent electrical properties of 44.26 cm^2^V^−1^s^−1^ mobility, 92 mV/dec. subthreshold swing (SS), 0.04 V hysteresis, and 3.93 × 10^10^ ON/OFF ratio, which are superior to those of the reported ITZO TFTs. In addition, incident light coupled with tunable photon energy is introduced to monitor the leakage current of various *T*_ITZO_ devices. The OFF-current results demonstrate that under the identical photon energy, many more electrons are photo-excited for the thicker *T*_ITZO_ TFTs. NBIS-induced *V*_th_ shift and SS deterioration in all TFTs are traced and analyzed in real time. As the *T*_ITZO_ thickens to near Debye length, the degree of degradation is exacerbated. When the thickness further increases, the notorious instability caused by NBIS is effectively suppressed. This study provides an important research basis for the application of ITZO-based TFTs in future displays.

## 1. Introduction

In the last two decades, thin-film transistors (TFTs) based on metal oxide semiconductors are one of encouraging low-input voltage electronic devices in transparent and flexible flat panel display (FPD) applications where traditional silicon-related TFTs are hard to match [1,2,3]. Among various metal oxide active layers, amorphous InGaZnO (a-IGZO)-dependent transistors have rapidly developed because they easily achieve high mobility (*μ*) of ~10 cm^2^V^−1^s^−1^ and long-term stability through creative approaches [4,5,6], including interface modification, doping engineering, and device structure adjustment. Nevertheless, their mobility is insufficient to satisfy the requirement of driving integrated circuits in ultra- and super-high-definition FPDs.

Due to the efforts of researchers, ternary and quaternary metal oxide semiconductors with different elements that regulate concentration have been designed in the pattern of permutation and combination. InSnZnO (ITZO), as an alternative candidate to active layers, can effectively contribute to a high *μ* of ~30 cm^2^V^−1^s^−1^ [7]. Simultaneously, other electrical parameters, including threshold voltage (*V*_th_) and subthreshold swing (SS), have also been substantially improved in comparison with those of a-IGZO TFTs. The possible reasons for enhancing the *μ* of ITZO-based TFTs are commonly attributed to two scenarios. One scenario is where the atomic radius of Sn is 1.40 Å, which is larger than that of Ga (1.26 Å). As a consequence, the average distance between the Sn atom and its adjacent In atoms in the ITZO lattice is shorter than that between Ga and In atoms in the IGZO lattice with the identical element content [8]. In addition, increasing the overlap probability of Sn 5s and In 5s atomic orbitals is beneficial because it raises the *μ* along the conduction band (*E*_C_). The other is because the electron effective mass in the ITZO system is slightly lighter than that in a-IGZO [8], contributing to the increase in field-effect mobility.

Although much research has been devoted to the development of metal oxide TFTs, device stability is still an issue, particularly under the photo-excitation conditions, which restrict the process of industrialization. In terms of a-IGZO materials, despite incident light energy being lower than the band gap of a-IGZO [9], leakage of current is still observed and defects can be found. Moreover, new defect states are created in the IGZO bulk, even along the contacted interfaces. However, the photo-assisted negative bias stress (NBIS)-caused performance deterioration can be effectively avoided via test intervention [10], anion introduction [11], and treatment temperature regulation [12,13]. On the other hand, the NBIS-resulting *V*_th_ shift and SS degradation can be quantitatively analyzed by designing the oxide semiconductor thickness. In previous studies [14], we explored the dark state stabilities under test temperatures and drain current stress in a-ITZO TFTs. We therefore investigated how the ITZO thickness influences photo-excitation on leakage current and negative bias instability in the corresponding TFTs.

In this regard, we first examined how ITZO TFTs with different thickness (*T*_ITZO_) are prepared. The electrical parameters of transfer curves for all devices were extracted and compared with the published ITZO-based TFT performance. We found that the electrical characteristics obtained in this study are generally superior to others. More importantly, the impacts of photo-excited OFF-current increase and NBIS-provoked instability in *T*_ITZO_-varied TFTs were systematically investigated.

## 2. Experimental Methods

To construct the ITZO TFTs with various *T*_ITZO_, the glass substrate was ultrasonically treated with acetone, isopropyl alcohol, and deionized water for 5 min, separately. The chromium film was fabricated on the treated glass and was dry etched to form the bottom electrode. The 150 nm thick SiO_2_ gate insulator was then using utilizing plasma-enhanced chemical vapor deposition (PECVD). In the case of the active layers, DC magnetron sputtering technology was adopted. The sputtering parameters, which included a deposition power of 60 W, operation pressure of 1 Pa, working gases of Ar and O_2_ with ratio of 15/15 sccm, and sputtering temperature of 30 °C, were fixed. In addition, the deposition duration was adjusted. Consequently, the ITZO films with the *T*_ITZO_ of 25, 45, 75, and 100 nm were tailored. After the pattern process, the geometric factor of length–width ratio was 20:50 μm as a result of the identical design specification. The SiO_2_ film (200 nm) was deposited again using PECVD to form an etch-stopper layer. The ITO source and drain electrodes were sequentially fabricated with the help of the sputtering method and etching technology. The SiO_2_ passivation layer was finally deposited to obtain bottom-gate top-contact ITZO TFTs. Subsequently, the devices were thermally heated in N_2_ at 350 °C for 1 h. In addition, the photo-excitation OFF-current results were investigated under illumination at wavelengths in the region of 400–530 nm. With regard to the operation conditions of NBIS evaluation, the wavelength, gate voltage, and stress duration were 460 nm, −20 V, and 10^4^ s, respectively. The current–voltage (I–V) response was measured using an Agilent 4156C semiconductor parameter analyzer.

## 3. Results and Discussion

**Figure 1:**
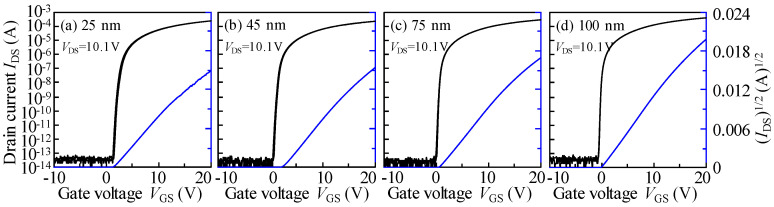
Transfer properties of (**a**) 25, (**b**) 45, (**c**) 75, and (**d**) 100 nm *T*_ITZO_-varied TFTs measured at *V*_DS_ = 10.1.

Figure 1 displays the transfer properties of ITZO thickness-varied TFTs measured at *V*_DS_ = 10.1 V. The corresponding electrical parameters calculated from the forward scan are listed in Table 1. With respect to the TFT with 25 nm *T*_ITZO_, outstanding electrical properties were observed: *μ*_sat_ of 34.73 cm^2^V^−1^s^−1^, *V*_th_ of 1.95 V, ON/OFF ratio of 2.38 × 10^10^, hysteresis Δ*V*_H_ of 0.17 V, and SS of 206 mV/dec. When the *T*_ITZO_ increased to 45 nm and continued to thicken to 100 nm, the *μ*_sat_ slightly increased to 37.61 cm^2^V^−1^s^−1,^ and gradually changed to 44.26 cm^2^V^−1^s^−1^. Additionally, as the *T*_ITZO_ increased, the *I*_D_ rose. Considering the relationship between *I*_D_ and *μ*_sat_ [15], the improvement in the *μ*_sat_ contributed to the increase in the ON current. The *V*_th_ negatively changed to 1.13 and −0.25 V, which correspond to 45 and 100 nm *T*_ITZO_ devices, respectively, as a result of the increase free carrier concentration in the thicker *T*_ITZO_. The ON/OFF ratio reached more than 10 orders of magnitude and the hysteresis is almost negligible irrespective of the *T*_ITZO_. The SS of 92 mV/dec. was obtained when the *T*_ITZO_ thickened to 100 nm. The SS value is a standard criterion of the total defect densities in the active layer and its adjacent interfaces [16]. This value suggests that high quality ITZO layer and front- and back-interfaces were designed in this work. Furthermore, compared with the performance of reviewed ITZO TFTs [17,18,19,20,21,22,23], as tabulated in Table 2, the 100 nm *T*_ITZO_ device in this study is superior.

**Figure 2:**
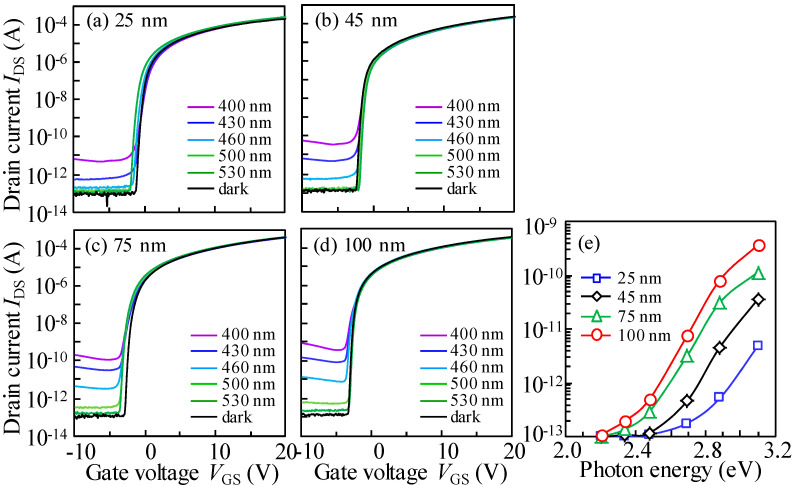
Variation in the OFF-current of the transfer curves in the reverse scan for TFTs with the *T*_ITZO_ of (**a**) 25, (**b**) 50, (**c**) 75, and (**d**) 100 nm under photo-excitation with various wavelengths, and (**e**) the corresponding OFF-current variation as a function of incident photo-energy.

The typical transfer curves of ITZO-varied devices were recorded from ON- to OFF-current direction under the external light exposure and *V*_DS_ bias of 20.1 V, as described in Figure 2a–d. In general, the OFF-current is dependent on the incident photon energy and the *T*_ITZO_, as summarized in Figure 2e. For the 25 nm *T*_ITZO_ TFT, when the incident light energy was greater than 2.70 eV, the photo-excited OFF-current slightly increased. This increase depends on the photon energy of incident light. As the *T*_ITZO_ s increased to 45 nm, high OFF-current was obtained, even under the identical photon energy of 2.70 eV. When the *T*_ITZO_ was further thickened to 75 and 100 nm, the photon energy of excited OFF-current gradually reduced to 2.48 and 2.34 eV, respectively. These phenomena can be interpreted as follows: for the channel layer with *T*_ITZO_ of 25 and 45 nm, the high-density defect states occupy the valence band maximum (*E*_V_), which is ~2.70 eV away from the conduction band (*E*_C_), similar to the results of a-IGZO devices [24]. In terms of the *T*_ITZO_ at 75 and 100 nm, given the long deposition duration in the sputtering chamber, the ITZO films suffer from strong plasma bombardment. A possible correction is the generation of more defect states located in the positions of 2.48 or 2.34 eV away from the *E*_C_. With regard to a-IGZO, the high density of oxygen vacancy (V_O_) defects with a width of ~1.5 eV is located near the *E*_V_ [25]. We therefore think that the long-term bombardment effect results in much higher densities of V_O_ defect states with the increased energy width, and gradually broadens with the increase in *T*_ITZO_.

**Figure 3:**
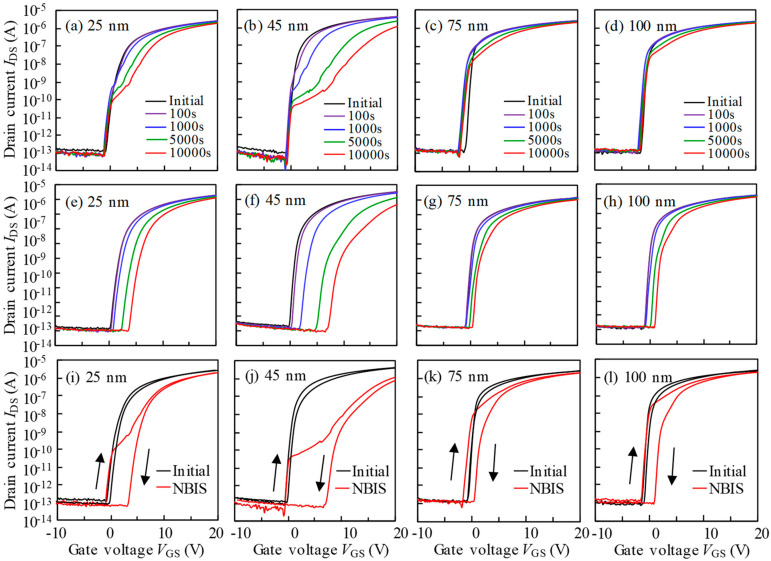
Evolution of transfer curves of TFTs with the *T*_ITZO_ of (**a**) 25, (**b**) 50, (**c**) 75, and (**d**) 100 nm in the forward scan, and the devices with thickness of (**e**) 25, (**f**) 50, (**g**) 75, and (**h**) 100 nm in the reverse scan as a function of NBIS duration. The whole transfer curves in the beginning and after NBIS stress of TFT devices with the *T*_ITZO_ of (**i**) 25, (**j**) 50, (**k**) 75, and (**l**) 100 nm, respectively.

**Figure 4:**
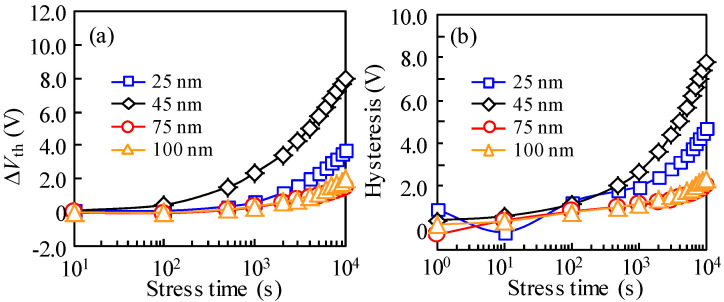
(**a**) The *V*_th_ variation in the reverse scan and (**b**) hysteresis change as a function of stress duration.

To further explore the collaborative effect of photo-excitation and negative bias on the stability of *T*_ITZO_-varied TFTs, we conducted a routine NBIS investigation. Regarding the forward scan (Figure 3a–d), the transfer curves of 25 nm *T*_ITZO_ device presented a positive *V*_th_ shift associated with SS decay. The NBIS-caused situation showed a progressive deterioration with increasing stress duration. A similar observation was made in the 45 nm *T*_ITZO_ case. Moreover, these phenomena were further amplified. With the increase in *T*_ITZO_, conditions relating to the positive movement of *V*_th_ and the degradation of SS value remarkably recovered. In For100 nm *T*_ITZO_ TFT, the instability that originated from NBIS was considerably suppressed. In the subsequent reverse scan (Figure 3e–h), the SS decay phenomenon was then generally restored. For the thin 25 nm *T*_ITZO_ particularly, the *V*_th_ positively changed 3.72 V with a stable SS. When the *T*_ITZO_ slightly thickened to 45 nm, the range of the *V*_th_ positive shift extended to 8.00 V, and the signs of SS decline were still found after a 5000 s stress duration. In addition, as the channel continued to thicken to 75 and 100 nm, similar phenomena were still observed. The changes in *V*_th_ drift reduced to 1.47 and 1.98 V, as plotted in Figure 4a, and we observed a tiny SS fluctuation.

**Figure 5:**
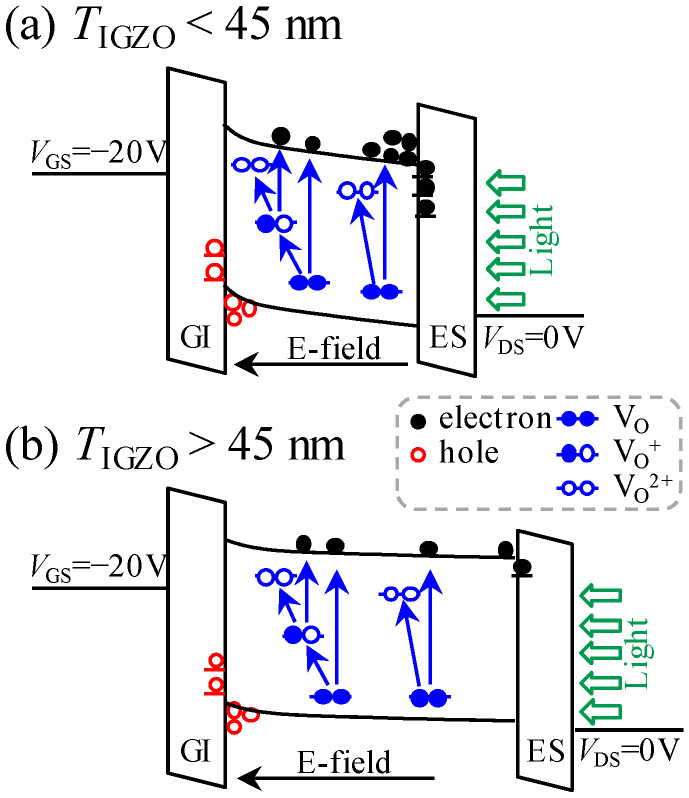
Diagrammatic sketch of NBIS-provoked instabilities in the devices with (**a**) *T*_ITZO_ < 45 nm and (**b**) *T*_ITZO_ > 45 nm.

Based on the results of photo-excited OFF-current and our previous research [13], we confirmed that the photo-generated holes and electrons drifted and were captured at the front- and back-interfaces under the action of the negative *V*_GS_-induced electric field, and the new donor-like defect states, such as V_O_^+^/V_O_^2+^, were created at the Fermi level around the turn-on voltage. In order to visually compare the role of NBIS on the hysteresis of *T*_ITZO_-varied TFTs, the transfer curves that were scanned from two different directions were combined (Figure 3i–l), and the corresponding hysteresis was quantitatively calculated (Figure 4b). For the 25 nm *T*_ITZO_ TFT, the combined actions of the created defect states and the captured photo-generated holes resulted in SS decay and the negative shift in *V*_th_ for the curves when *V*_GS_ was scanned from −10 to 20 V. After the forward scan, the V_O_^+^/V_O_^2+^ defect states stabilized due to a high *V*_GS_. However, the trapped holes at the front-interface were hard to desorb. Simultaneously, the electrons excited from deep level states were driven by electric field and trapped at the back-interface. The number of captured electrons increased with the extension of NBIS duration, leading to the positive shift in curves without SS deterioration. In combination with all the factors, a 4.69 V hysteresis was obtained after a duration of 10^4^ s NBIS. The above analysis is also applicable to the 45 nm *T*_ITZO_ device, where all the factors amplified and intensified, including electron/hole trapping and V_O_^+^/V_O_^2+^ creation, which facilitated a severe hysteresis of 7.78 V, as described in Figure 5a. We found that the *T*_ITZO_ of 45 nm is a point of inflection because it is close to the Debye length (~40 nm), which has the longest transmission path [16]. When the *T*_ITZO_ thickened to 75 and 100 nm, far from the Debye length, it was difficult for the excited electrons to be trapped at the back-channel interface, thereby resulting in the slight movement of the reverse-swept transfer curves. At the same time, these free electrons stabilized the created V_O_^+^/V_O_^2+^ defects and recombined some of the photo-generated holes, which is sketched in Figure 5b, consequently contributing to the relatively small hysteresis values of 1.99 and 2.36 eV, respectively.

On the basis of all the results, the high-performance ITZO TFTs were produced by tailoring the *T*_ITZO_. For traditional a-IGZO TFTs, their overall performance is commonly improved by introducing other elements, optimizing the post-treatment process, and adjusting the test conditions. However, these devices, especially for a *T*_ITZO_ of 100 nm, are successfully implemented with perfect initial electrical performance and relative long-range NBIS stability without further modification and treatment.

## 4. Conclusions

The sputtering processed ITZO films with different *T*_ITZO_ for TFT application were reported. By using the traditional bottom-gate top-contact device architecture, the ITZO-based TFTs exhibit outstanding electrical properties, specifically with regard to the 100 nm *T*_ITZO_ case, which are generally superior to the performance of reviewed ITZO TFTs. Furthermore, the roles of photo-excited OFF-current and NBIS-provoked instability in *T*_ITZO_-varied TFTs were determined. The leakage current analysis revealed that many more electrons are generated for the thicker *T*_ITZO_ device. When the *T*_ITZO_ is close to Debye length, we used NBIS-caused hysteresis to analyze the instability. NBIS-induced stability deteriorations are remarkably improved in the thicker *T*_ITZO_ TFTs. This study demonstrates the value of *T*_ITZO_ for tailoring photo-induced degradation mechanisms in the devices and facilitates the commercialization of high-performance ITZO TFT-based FPDs.

## Figures and Tables

**Table 1:** The extracted parameters of *T*_ITZO_–varied TFTs in the forward scan.

Thickness (nm)	25	45	75	100
*μ*_sat_ (cm^2^∙V^−1^∙s^−1^)	34.73	37.61	41.63	44.26
*V*_th_ at *I*_DS_ = 1 nA (V)	1.95	1.13	0.42	−0.25
ON/OFF ratio (10^10^)	2.38	2.22	2.87	3.93
Hysteresis Δ*V*_H_ (V)	0.17	0.16	0.08	0.04
Subthreshold swing (mV/dec.)	206	167	131	92

**Table 2:** Comparison of the electrical characteristics of various *T*_ITZO_ devices and other ITZO-based TFTs in the literature.

Preparation of ITZO	*T*_ITZO_ (nm)	*μ*_sat_ (cm^2^∙V^−1^∙s^−1^)	*V*_ON_ (V)	*I*_OFF_ (A)	ON/OFF Ratio	SS (mV/dec.)	Reference
DC sputtering	30	31.19	−0.93	10^−14^	−	153	[17]
RF sputtering	50	28.97	−	10^−13^	2.64 × 10^7^	~200	[18]
DC sputtering	−	30.90	0.97	10^−14^	−	~210	[19]
Spin-coating	41	9.80	−1.50	10^−7^	1.30 × 10^3^	~2300	[20]
−	50	37.20	−	10^−12^	~1 × 10^7^	~930	[21]
Co-sputtering	25	24.60	−0.40	10^−14^	10^9^	~120	[22]
Co-sputtering	60	10.40	2.10	10^−12^	2.30 × 10^7^	93	[23]
RF sputtering	100	44.26	−0.25	10^−14^	>10^10^	92	This work
